# Deletion viral genome diversity among bovine viral diarrhea virus (BVDV) 1a and 1b strains

**DOI:** 10.1186/s12985-025-02773-z

**Published:** 2025-07-14

**Authors:** David J. Holthausen, Darrell O. Bayles, John D. Neill, Rohana P. Dassanayake, Shollie M. Falkenberg, Harish Menghwar, Eduardo Casas

**Affiliations:** 1 Ruminant Diseases and Immunology Research Unit, Agricultural Research Service, National Animal Disease Center, USDA, Ames, IA 50010 USA; 2 Infectious Bacterial Diseases Research Unit, Agricultural Research Service, National Animal Disease Center, USDA, Ames, IA 50010 USA; 3 Department of Pathobiology, College of Veterinary Medicine, Animal Health Research, Auburn University, Auburn, AL 36849 USA; 4 Oak Ridge Institute for Science and Education (ORISE), ARS Research Participation Program, Oak Ridge, TN 37831 USA

**Keywords:** Bovine viral diarrhea virus (BVDV), *Pestivirus*, Nonstandard viral genomes (NSVGs), Deletion viral genomes (DelVGs), Viral evolution

## Abstract

**Background:**

Bovine viral diarrhea virus (BVDV) is a pervasive respiratory pathogen of economic concern for the cattle industry. Transplacental infection results in abortion or the establishment of a tolerant and persistent viral infection. Deletion viral genomes (DelVGs) are naturally occurring products of the viral replication process. These deletion viral genomic transcripts are generated with truncations of various sizes that severely impede or prevent self-replication. DelVGs have been implicated in the establishment of viral persistence.

**Methods:**

We used a bioinformatic pipeline to discover the presence of BVDV DelVGs. These DelVGs were identified via analysis of Illumina MiSeq reads from 74 BVDV1 field isolates from two closely related subgenotypes and from an *in vitro* passage of a BVDV1a virus at two different multiplicities of infection (MOI).

**Results:**

After the identification of DelVGs, we assessed their phylogenetic linkage to begin elucidating potential roles in the viral life cycle and persistence. BVDV1a viruses queried generate significantly more DelVGs, with 52% of 5’ and 3’ junctions occurring in the core/capsid (C) region and a major NS2-NS5B deletion species. In contrast, the BVDV1b viruses generated significantly fewer DelVGs, especially a reduction in C region deletions. *In vitro* passaging of the BVDV1a Singer virus demonstrated that MOI significantly impacts the generation of DelVGs, with higher MOIs generating more DelVGs and a different deletion profile.

**Conclusions:**

Here, we report that the BVDV1a and BVDV1b subgenotypes generate diverse species of DelVGs. These DelVGs may play key roles in BVDV evolution and the establishment of persistence during transplacental infection.

**Supplementary Information:**

The online version contains supplementary material available at 10.1186/s12985-025-02773-z.

## Background

Bovine viral diarrhea virus (BVDV) is a single-stranded positive-sense RNA virus of the genus *Pestivirus* of the family *Flaviviridae* and is a pathogen that causes substantial economic loss in the cattle industry. While most acute respiratory infection cases are mild, BVDV is a known immunosuppressor that can predispose and worsen secondary infections, contributing to bovine respiratory disease complex (BRDC), which presents as bacterial and viral pneumonia [1, 2]. Most consequentially, however, BVDV is associated with significant reproductive losses [3]. Transplacental infection often results in abortion. However, if infection occurs during days 60-120 of gestation, calves become tolerant and persistently infected (PI), acting as a reservoir for continuous infection in a herd after they are born [3–6]. BVDV-associated morbidity and mortality have been estimated to cost the US beef industry an estimated $2 billion per year as of 2016 and are especially problematic at centers of herd commingling, such as feedlots [7]. The combination of fetal infection-induced abortion, viral tolerance, and immune suppression is a significant challenge to the livestock industry.

The *Pestivirus* genus consists of 11 members, including BVDV1 and BVDV2, classical swine fever (CSFV), border disease (BDV), pronghorn antelope pestivirus (PHV), Porcine pestivirus (BuPV), Giraffe pestivirus, HoBi-like Pestivirus (HoBi), Aydin-like pestivirus, Rat pestivirus (NrPV), and atypical porcine pestivirus (APPV). Recently, the members of the family were taxonomically reclassified into 19 different species [8]. BVDV1 and BVDV2 are classified as *Pestivirus bovis* and *Pestivirus tauri*, respectively, and the highly related HoBi-like *Pestivirus* is *Pestivirus brazilense*. The approximately 12.3 kb genome of BVDV has high genetic diversity. BVDV-infected PI calves are known to harbor a high diversity of viral quasispecies [9]. By phylogenetic analysis, the 2 genotypes, BVDV1 and BVDV2, are currently divided into 21 subgenotypes (BVDV1a-u) and 4 subgenotypes (BVDV2a-d), respectively [10]. Two biotypes, cytopathic (CP) and noncytopathic (NCP), of BVDV exist. Among these two biotypes, only NCP viruses establish persistent infection in the fetuses of cows infected prior to the maturation of the fetal adaptive immune system [11]. Like other flaviviruses, the BVDV genome is a long single open reading frame (ORF) that is not polyadenylated, has no 5’ cap, encodes 5’ and 3’ untranslated regions (UTRs), and is a polyprotein that is post translationally cleaved. The enveloped particles contain four structural proteins: a capsid/core (C) protein and three envelope glycoproteins, Erns unique to pestiviruses, E1, and E2 [12–14]. The remaining encoded nonstructural proteins are the unique n-terminal NPro auto protease, p7, NS2-3 proteases, NS4 A, NS4B, NS5 A, and NS5B RNA-dependent RNA polymerase (RdRp) [15, 16]. While BVDV genomic diversity and protein function are areas of active discovery, the nature of nonstandard viral genome (NSVG) generation during viral replication has not been previously assessed or reported.

Nonstandard viral genomes (NSVGs) are a naturally occurring phenomenon of positive- and negative-sense RNA viral replication. Originally described as defective viral genomes (DVGs), the generation of severely truncated genomic variants that cannot self-replicate has been proven to play critical roles in the dynamics of virus‒host interactions and is speculated to be critical for maintaining a fit viral population [17–19]. The generation of NSVGs was first linked to virus growth at high MOIs in the 1940s in an influenza system [20–22]. These genomes are packaged into viral particles, creating defective interfering particles (DIPs) with three well-characterized functions [23]. Nonstandard viral genomes and DIPs interfere with the replication of standard-length viruses by competing for replication machinery, aiding in the establishment of viral persistence, and acting as a primary trigger of the host innate immune response [24–27]. Nonstandard viral genomes are strong inducers of the host type I and type III interferon response and primers of antigen-presenting cells; however, this is chiefly attributed to copyback viral genomes (CbVGs) generated by negative-sense viruses [28–30]. As such, they are an active area of antiviral and vaccine development [31–34]. In contrast, positive-sense viruses are associated primarily with generating deletion viral genomes (DelVGs) [18].

The deletion viral genome is characterized by normally single large internal truncations in the viral genome where one or more genes required for self-propagation are rendered nonfunctional [35, 36]. While many viral proteins are involved in the generation of DelVGs and other types of NSVGs, the role of RdRp and its ability to modulate its fidelity have been the most scrutinized and studied [37, 38]. Many flaviviruses, including dengue, hepatitis C, and West Nile, have been shown to generate DelVGs [39–41]. The DelVGs generated by flaviviruses have been implicated in impacting virulence and establishing viral persistence [18]. In this study, we sought to characterize the NSVGs generated by the two most abundantly circulating, studied, and closely related BVDV subgenotypes, BVDV1a and 1b. We also hypothesized that MOI-dependent growth conditions may impact the frequency and nature of NSVGs generated during BVDV replication *in vitro*. Bioinformatic tools have been available for the last decade for querying viral genome sequencing data to detect NSVGs [42, 43]. In this study, we chose to use a recently updated Viral Opensource DVG Key Algorithm 2 (VODKA2) pipeline for the identification of NSVGs within next-generation sequencing (NGS) datasets [44]. Here, we queried the NGS genomic sequencing data of more than 70 unique BVDV1a and BVDV1b isolates from the last 30 plus years, as well as an *in vitro* passage experiment of the Singer BVDV1a variant, to better understand any phylogenetic or dynamic linkages or variations among highly related and diverse BVDV1a and 1b strains.

## Methods

### Virus strains and RNA extraction

Seventy-four field isolates from clinical samples submitted to the National Animal Disease Center from the 1980s through the 2010s, comprising 41 BVDV1a isolates and 33 BVDV1b isolates, were selected for this study. BVDV strains were isolated from these samples as previously described [45]. Briefly, tissue homogenates, buffy coats, or serum samples were inoculated onto Madin-Darby bovine kidney cells (MDBKs) (Gibco MEM 11095 supplemented with 10% filtered and heat-inactivated FBS (HI-FBS)) for 1 hour at 37 °C in a 5% CO_2_ incubator. The cells were washed to remove the inoculum, and fresh media containing 10% fetal bovine serum (confirmed BVDV and BVDV antibody-free) was added and returned to the incubator for 4‒5 days prior to collection. Inoculation onto MDBKs confirms viral viability and improves probability of viral isolation. In the absence of cytopathic effects (CPE), the cells were fixed and stained as previously described [46]. BVDV RNA was extracted via QiaCube Viral RNA kits according to the manufacturer’s instructions (Qiagen, Valencia, CA), as we have previously described [47]. BVDV1a, BVDV1b, and BVDV2a genomic sequences were obtained from NCBI GenBank as references for phylogenetic comparison (Supplemental Table 1).

### Sequencing and phylogenetic analysis

Whole-genome sequencing was conducted as previously described [45]. Briefly, whole-genome sequencing was conducted via cDNA synthesis from viral RNA with 20 base primers of known sequence with 8 random bases at the 3’-end of the primer, yielding barcode identifiable cDNAs that were amplified via primer-specific polymerase chain reaction (PCR) and sequenced via the MiSeq platform (Illumina, Inc., San Diego, CA). Sequencing depth was standardized across all sample sets. Viral genomes were assembled via de novo and reference-assisted assembly via SeqManNGen Version 12 (LaserGene, Inc., Madison, WI). Initial subgenotype classification of the assembled viruses was conducted via alignment of all 5’ UTR sequences, as is commonly employed [46, 48]. Phylogenetic analysis for subgenomic relatedness was completed via whole-genome alignment. The Molecular Evolutionary Genetics Analysis 7 (MEGA7 version 10.2.2) software tool’s Clustal W alignment and the UPGMA method were utilized to generate phylogenetic trees with branch support estimated via 1000 bootstrap replicates and the Poisson correction method to calculate the evolutionary distances [49].

### Viral Opensource DVG Key Algorithm 2 (VODKA2) deletion viral genome analysis

The VODKA2 pipeline was run with only the minimal modifications needed for the local runtime environment [44]. The VODKA2 analysis workflow requires a specified reference sequence. For this study, we used the following NCBI accessions as our reference genomes for the different groups of BVDV isolates that were sequenced (BVDV1a - MH133206.1 Bovine viral diarrhea virus 1 strain Singer Arg, complete genome; BVDV1b – MH490943.1 Bovine viral diarrhea virus 1b strain BVDV BJ-2016, complete genome). The genomes were formatted into a large Bowtie2 database via the “genomefa_to_newfasta_del_v2.pl” VODKA2 script with the following parameters: the length of the reference genome in base pairs (bp), the sequencing read length was 151 bp, and the gap size was 10 bp. After the Bowtie2 database was created, the paired-end Illumina MiSeq reads for each isolate were analyzed against the appropriate database via the VODKA2_analysis_setup.sh script. The DelVG “species” consists of reads with the same theoretical deletion size but with 5’ and 3’ junction reads within a range of ±5 nucleotides. For each viral strain, species with only a single read were removed from the analysis.

### In vitro viral passage

The well-studied BVDV1a CP Singer strain was chosen for the passage experiment. To purify and titer the virus, 100 µl of a 1:10 serial dilution was inoculated onto a monolayer of bovine turbinate (BTu) cells (Gibco MEM 11095 supplemented with 10% filtered and heat-inactivated FBS (HI-FBS), L-glutamine, and antibiotic-antimycotic). BTu cells were used instead of MDBK cells because plaques are more easily visualized for plaque purification in BTu cells in comparison to MDBKs. The infected monolayers were incubated for 4 to 6 hours at 37 °C and shaken gently several times during that period to aid in absorption. The infection media was aspirated and overlaid with sterile 1% UltraPure Low Melting Point Agarose (Thermo Fisher 16520100) in growth media (Gibco MEM 11935046, supplemented with 10% filtered and heat-inactivated FBS (HI-FBS), L-glutamine, and antibiotic-antimycotic), allowed to rest at room temperature for 15 minutes and then placed in a 37 °C humidified incubator with 7.5% CO_2_ for 5-7 days for plaque formation. Plaques were picked and aliquoted into 250 µl of MEM growth media without fetal bovine serum, passaged and titered via the Reed and Muench method [50]. The viral titer was also confirmed via a previously described 90–368 BVDV PCR method [51]. After titration, MOIs of 10 and 0.1 were passaged onto a new monolayer of BTu cells, which were agar overlaid, and plaques were allowed to form over 7 days, like the first passage. Plaques were picked, titered via the 90–368 PCR method, and passaged again for a total of 8 passages. At the time of plaque picking after each passage at 2 different MOIs, viral RNA was extracted, cDNA was generated via Superscript III reverse transcriptase (Thermo Fisher Scientific, Inc.), and whole-genome sequencing was conducted as previously described and detailed above [45, 52].

### Statistical analysis

Statistical analysis tests were performed as indicated in each figure legend via GraphPad Prism version 10.2.0 (GraphPad Software, Inc.).

## Results

To assess whether BVDV viruses generate NSVGs, we selected 41 BVDV1a and 33 BVDV1b isolates, including some that have been previously sequenced by next-generation sequencing (NGS), for analysis [53–56]. The selected isolates were mapped against known BVDV1a (Singer Arg, GenBank ID: MH133206.1) or BVDV1b (BJ-2016, GenBank ID: MH490943.1) sequences to confirm sequence fidelity prior to VODKA2 pipeline analysis. The subtype and phylogenetic relationships of the analyzed strains were confirmed via complete genome sequence alignment of all the experimental isolates, as were the verified sequences of the BVDV1a, 1b, and 2a subtypes obtained from the NCBI GenBank (Supplemental Fig. 1 A, B).

### BVDV1a viruses generate deletion viral genomes with a high proportion in the core/capsid region

**Fig. 1:**
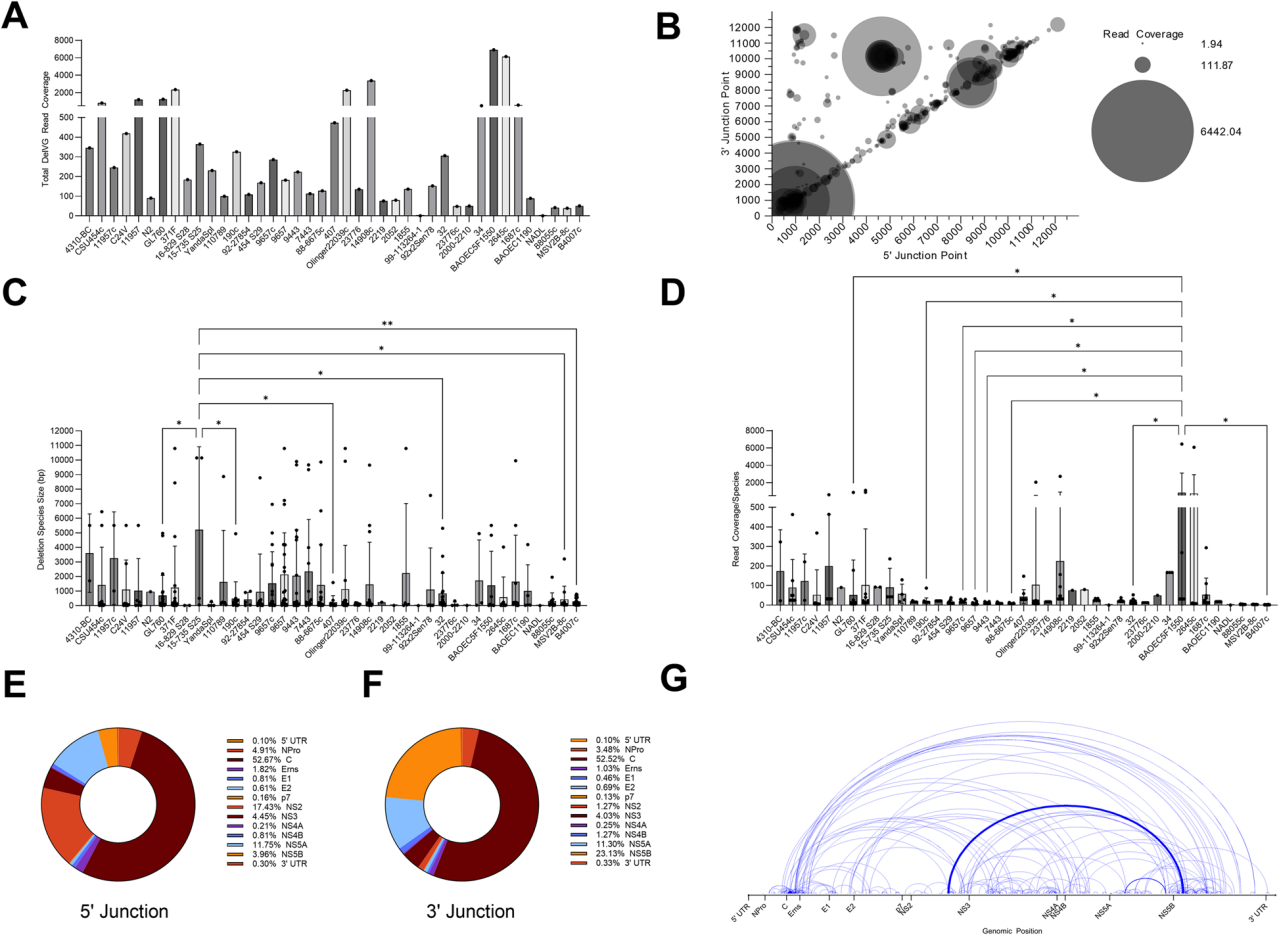
DelVG analysis of BVDV1a virus strains. **A** Total strain DelVG read coverage per 1 million standard viral reads. BVDV1a strains are ordered along the x-axis by full-genome Clustal W phylogenetic analysis via MEGA7 software. **B** 5’ and 3’ junction point analysis for all BVDV1a strains. The area of each data point correlates to the read coverage per 1 million standard viral reads. **C** Strain-specific read coverage per DelVG species per 1 million standard viral reads. BVDV1a strains are ordered along the x-axis by full-genome Clustal W phylogenetic analysis via MEGA7 software. Two-way ANOVA with Ŝídák multiple comparison test was performed for statistical analysis. Ns: p>0.05, *: p<0.05, **: p<0.005. **D** Strain-specific DelVG species sizes (bp). BVDV1a strains are ordered along the x-axis by full-genome Clustal W phylogenetic analysis via MEGA7 software. Two-way ANOVA with Ŝídák multiple comparison test was performed for statistical analysis. Ns: p>0.05, *: p<0.05. **E** Distribution of 5’ junction reads per BVDV1a gene feature. **F** Distribution of 3’ junction reads per BVDV1a gene feature. **G** Representative depiction of the 5’ and 3’ junction points of individual BVDV1a DelVGs. Each arc represents an individual DelVG species. The width of each arc is proportional to the read coverage (per 1 million standard viral reads) per DelVG species

The sequencing reads for the 41 BVDV1a isolates were assessed for DelVGs. Thirty-nine of the 41 isolates assessed included DelVG reads among the standard viral genome reads (Fig 1A). To determine whether there are any phylogenetic patterns in terms of the size of the deletions or quantity of reads within the BVDV1a subset, we grouped the strains by sequence alignment (Fig 1 C, D). While there were substantial differences in the deletion sizes between the BVDV1a strains, there was no difference in phylogenetic pattern. Higher incidences of deletion species occurred in a few subsets of the BVDV1a isolates, with one strain, BAOEC5 F1550, having more deletion species than 8 other strains. Across the BVDV1a isolates, deletions were found with 5’ and 3’ junction points across the entire length of the genome, although loci in the 900-base pair (bp), 4500-bp, and 9000-bp regions had much higher incidences of deletions (Fig 1B).

We sought to translate these loci along the genome into the different final protein products of the BVDV polyprotein and assess common deletions across the individual isolates. For BVDV1a, approximately 52% of the BVDV1a deletions occurred in the C region, where the 5’ and 3’ junction points are within the same gene feature (Fig 1E, F). In addition to 52% of the deletions occurring in the C region, there is also a hotspot for deletions where the 5’ junction point occurs in the NS2 region and the 3’ junction point is in the NS5B-RdRp region (Fig 1G). Across all 41 BVDV1a strains studied, 10 deletion species occurred in 3 or more of the strains (Table 1). Among these common deletion species, the two most common were the 974-987C and 920-936C deletions present in 12 strains (29%) and 10 strains (24%), respectively, accounting for a read coverage of more than 15,000 reads. The third most common deletion is the NS2-NS5B species, which was present in 8 strains (20%), where the 5’ junction point occurs at position 4648 and the 3’ junction point is at position 7168. Given the relative abundance of these deletion species among the strains, we were curious if the strains in which these species occur are highly related. The most common deletion species were mapped to the phylogenetic tree of the aligned BVDV1a strains (Fig 3 A). Among the 10 common deletion species, only 3, NPro-C 442-944, NPro 551-564, and NS4B 7825-7836, clustered with highly related BVDV1a strains. The other 7 deletion species, including the 3 most common, were broadly distributed among the BVDV1a strains.

**Table 1:**
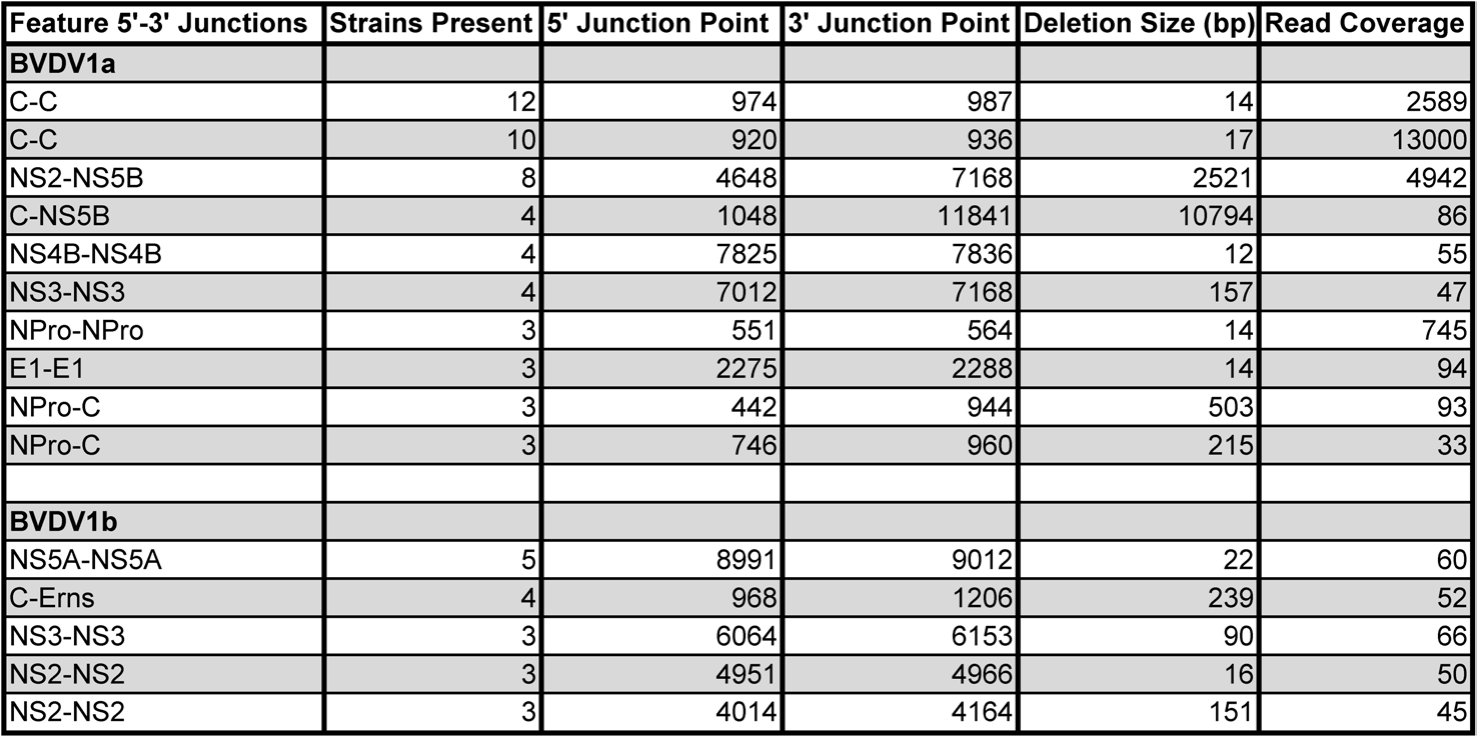
Most common deletion species (n ≥3 strains )

Common DelVG species determined by presence in 3 or more analyzed strains in BVDV1a and BVDV1b. From left to right: gene feature where 5' and 3' junctions occur; Number of strains DelVG species is present in; 5' junction point locus in genome; 3' junction point locus in genome; Size of DelVG in base pairs; Sum of read coverage across all strains present, normalized per 1 million standard viral reads

### BVDV1b viruses generate fewer capsid/core and overall deletion viral genomes

**Fig. 2:**
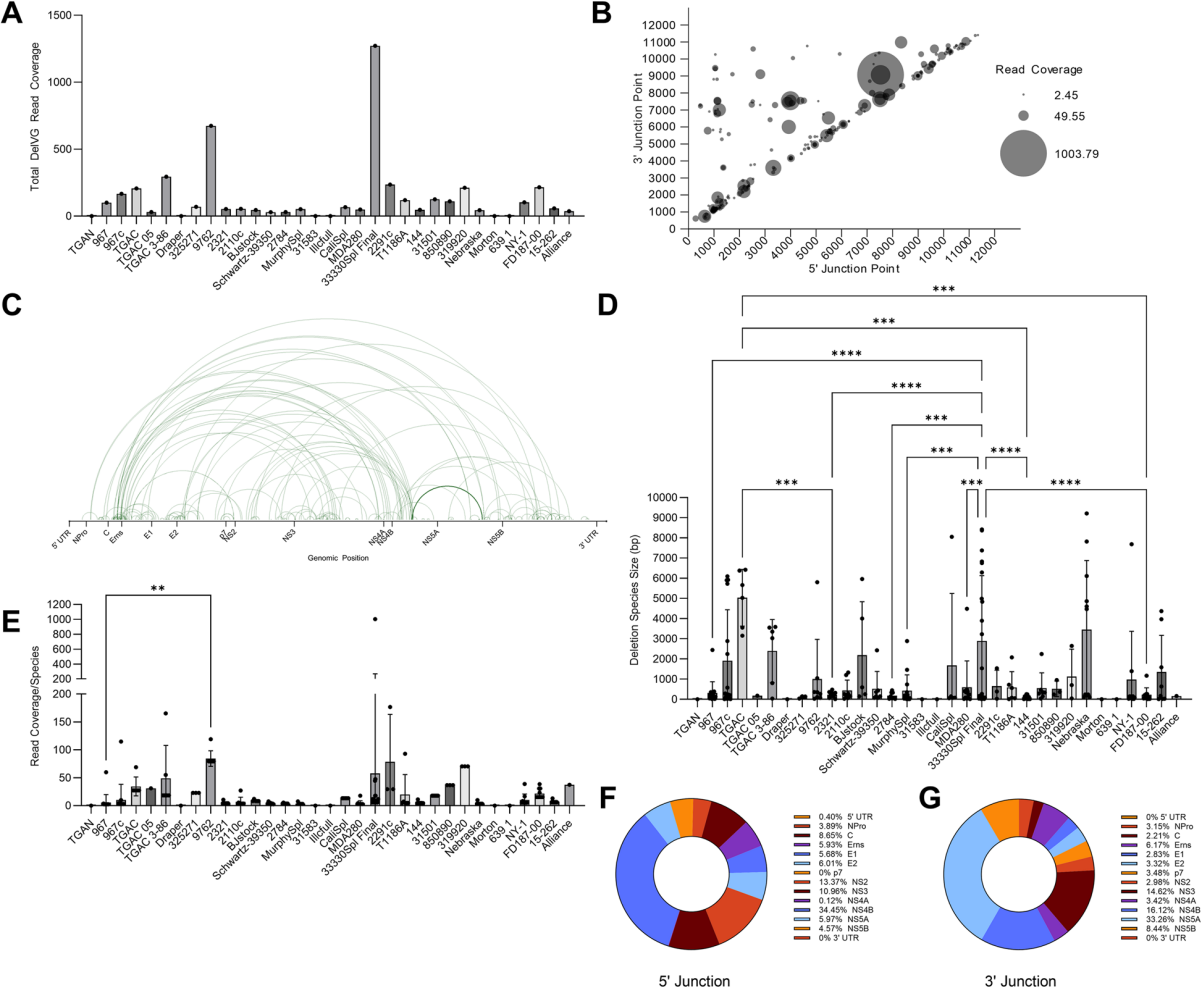
DelVG analysis of BVDV1b virus strains. **A** Total strain DelVG read coverage per 1 million standard viral reads. BVDV1b strains are ordered along the x-axis by full-genome Clustal W phylogenetic analysis via MEGA7 software. **B** 5’ and 3’ junction point analysis for all BVDV1b strains. The area of each data point correlates to the read coverage per 1 million standard viral reads. **C** Representative depiction of the 5’ and 3’ junction points of individual BVDV1b DelVGs. Each arc represents an individual DelVG species. The width of each arc is proportional to the read coverage (per 1 million standard viral reads) per DelVG species. (**D**) Strain-specific DelVG species sizes (bp). BVDV1b strains are ordered along the x-axis by full-genome Clustal W phylogenetic analysis via MEGA7 software. Two-way ANOVA with Ŝídák multiple comparison test was performed for statistical analysis. Ns: p>0.05, ***: p<0.0005, ****: p<0.0001. (**E**) Strain-specific read coverage per DelVG per 1 million standard viral reads. BVDV1b strains are ordered along the x-axis by full-genome Clustal W phylogenetic analysis via MEGA7 software. Two-way ANOVA with Ŝídák multiple comparison test was performed for statistical analysis. Ns: p>0.05, **: p<0.005. (**F**) Distribution of DelVG 5’ junction points per BVDV1b gene feature. (**G**) Distribution of DelVG 3’ junction points per BVDV1b gene feature

**Fig. 3:**
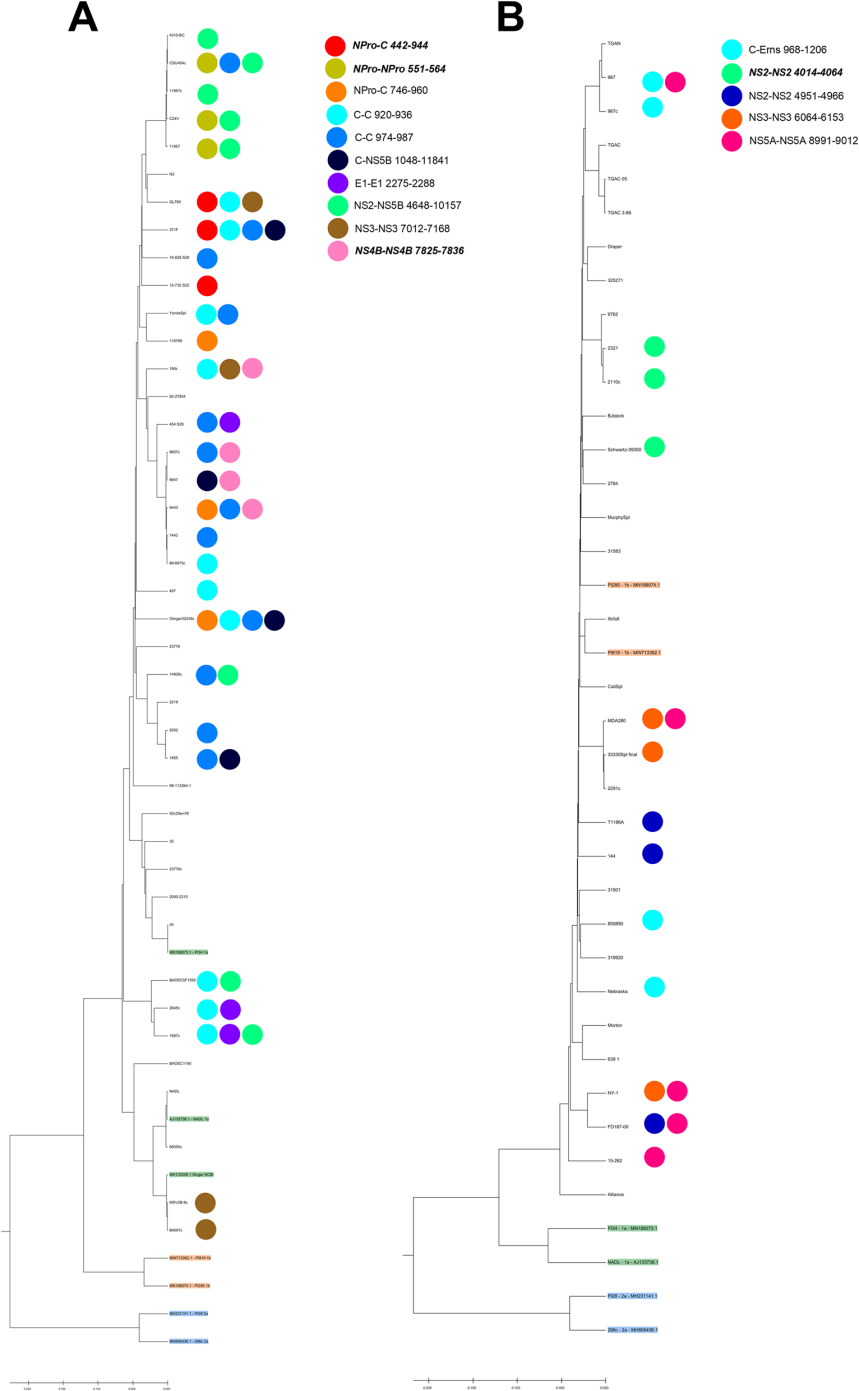
Phylogenetic assessment of the most common BVDV1a (**A**) and BVDV1b (**B**) deletion species. DelVG species found in 3 or more BVDV1 strains included. Further details are annotated in Table 1. The color-coded species are aligned with the virus strains they are present in. Phylogenetic dendrograms of BVDV1 strains generated via Clustal W alignment and the UPGMA method via MEGA7 software. Branch support was estimated via 1000 bootstrap replicates and the Poisson correction method to calculate the evolutionary distances. Verified comparative sequences from NCBI GenBank; BVDV1a (green), BVDV1b (orange), BVDV2a (blue). Bold and italics indicate species with phylogenetic clustering

**Fig. 4:**
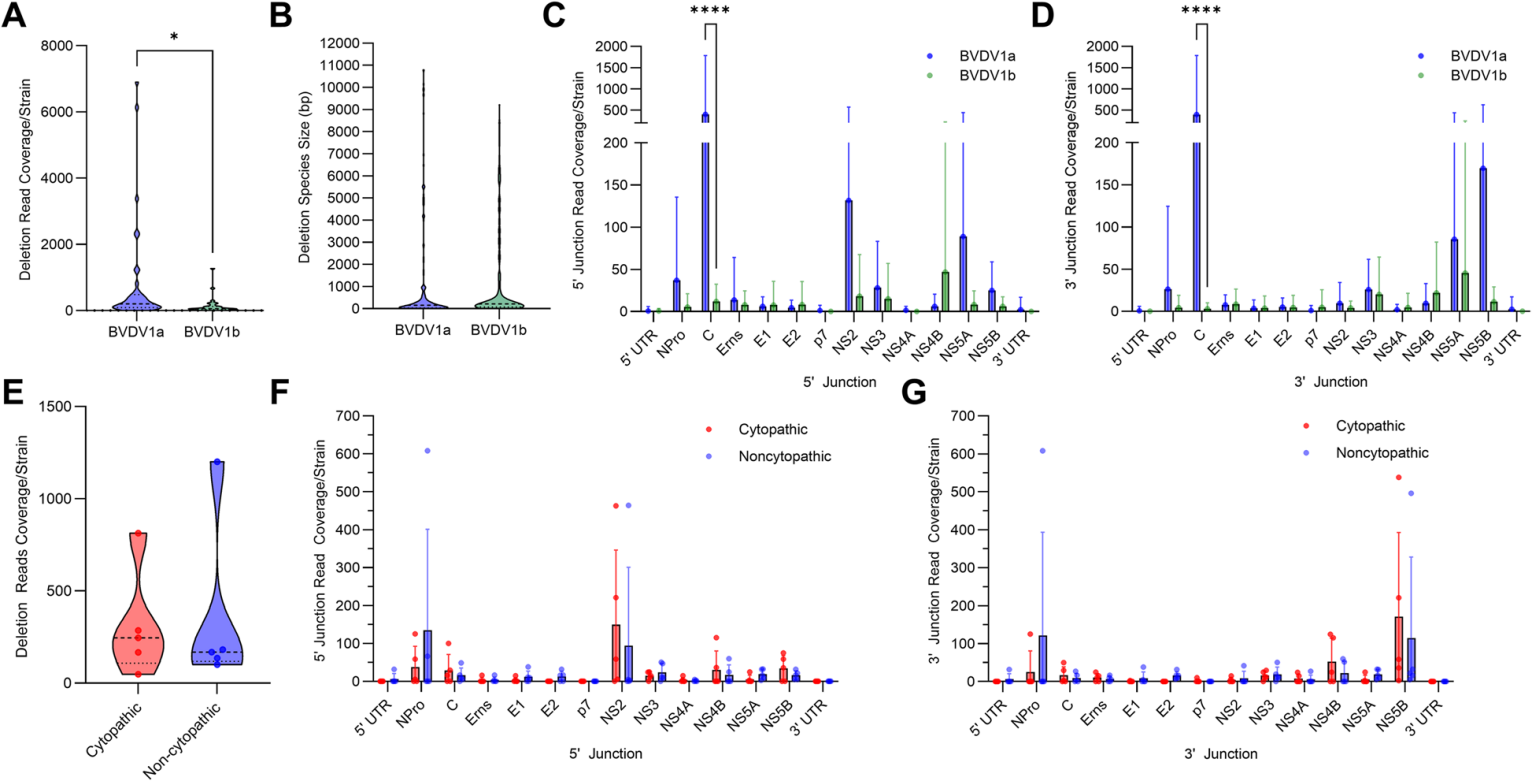
Comparative analysis of genotype and biotype DelVGs. **A** Total deletion reads (read coverage per million standard viral reads) among the BVDV1a and BVD1b strains. Unpaired t tests were performed for statistical analysis. *: p<0.05. **B** Size of each deletion species (base pairs) among all BVDV1a and BVDV1b strains sequenced. Data does not weigh reads per species. Unpaired t tests were performed for statistical analysis. There was no significant difference. Locations of the 5’ (**C**) and 3’ (**D**) junction points of all BVDV1a and BVDV1b DelVG reads (read coverage per 1 million standard viral reads). Two-way ANOVA with Ŝídák multiple comparison test was performed for statistical analysis. Ns: p>0.05, ****: p<0.0001. **E** Total deletion reads (read coverage per 1 million standard viral reads) among 5 pairs of CP (BVDV1a: 9657c, 11957c, CSU 454c, 23776c, BVDV1b: 967c) and NCP (BVDV1a: 9657, 11957, 454 S29, 23776, BVDV1b: 967) virus strains. A paired t test was performed for statistical analysis. There was no significant difference. Locations of the 5’ (**F**) and 3’ (**G**) junction points among 5 pairs of CP (BVDV1a: 9657c, 11957c, CSU 454c, 23776c, BVDV1b: 967c) and NCP (BVDV1a: 9657, 11957, 454 S29, 23776, BVDV1b: 967) DelVG reads (read coverage per 1 million standard viral reads). Two-way ANOVA with Ŝídák multiple comparison test was performed for statistical analysis. There was no significant difference

Among the 33 BVDV1b strains examined for NSVGs, DelVGs were present in 27, with 6 lacking any deletion reads (Fig 2A). In contrast to BVDV1a, BVDV1b does not have hotspots for deletion reads at the 900-bp, 4500-bp, or 9000-bp zones. While deletions are observed across the entire genome, BVDV1b has a deletion hotspot with a 5’ junction at approximately position 8000 and a 3’ junction at position 9000 (Fig 2B). This hotspot translates to a 5’ junction point in the NS4B region and a 3’ junction location in the NS5 A region (Fig 2C). Approximately one-third of BVDV1b-deletion 5’ junctions are at NS4B and 3’ junctions at NS5 A, with only 8% of BVDV1b-deletion 5’ junctions and 2% of BVDV1b-deletion 3’ junctions in the C region, in stark contrast to BVDV1a (Fig 2F, G). Furthermore, while deletion species sizes are comparable between BVDV1a and BVDV1b, BVDV1a viruses generate more deletion variants overall than BVDV1b viruses do (Fig. 4A, B). Unsurprisingly, this difference is directly attributable to a significant reduction in deletions in the C region (Fig 4C, D). However, like BVDV1a, there is no significant phylogenetic link among BVDV1b viruses in terms of the size of the deletion species or the frequency of the deletion species (Fig 2D, E). Among the BVDV1b strains analyzed, only 5 common deletion species were observed, with a maximum presence of 5 strains (15%) (Table 1). Among these 5 deletion species, only NS2 4014-4064 demonstrated any localization along the BVDV1b phylogenetic tree (Fig 3B). The other 4 common deletion species are distributed along the tree.

### No significant link between deletion viral genomes and biotype

While there is a distinct difference in the DelVG species generated by BVDV1 subgenotypes a and b, we were also interested in determining whether biotype contributes to differences in NSVGs generated during viral replication. BVDV is characterized into cytopathic (CP) and noncytopathic (NCP) biotypes based on whether they generate a cytopathic effect (CPE) *in vitro*. Five CP/NCP virus pairs (4 BVDV1a and 1 BVDV1b) were analyzed as a part of this study. There were no significant differences in the number of DelVGs between the CP and NCP virus pairs (Fig. 4E). There were also no significant differences in the 5’ junction or 3’ junction gene features between the biotype virus pairs (Fig. 4F, G). There was no correlation between biotype and any differences in the type of NSVG generated by BVDV, at least among the queried strains.

### The multiplicity of infection impacts the generation of deletion viral genome species in vitro

**Fig. 5:**
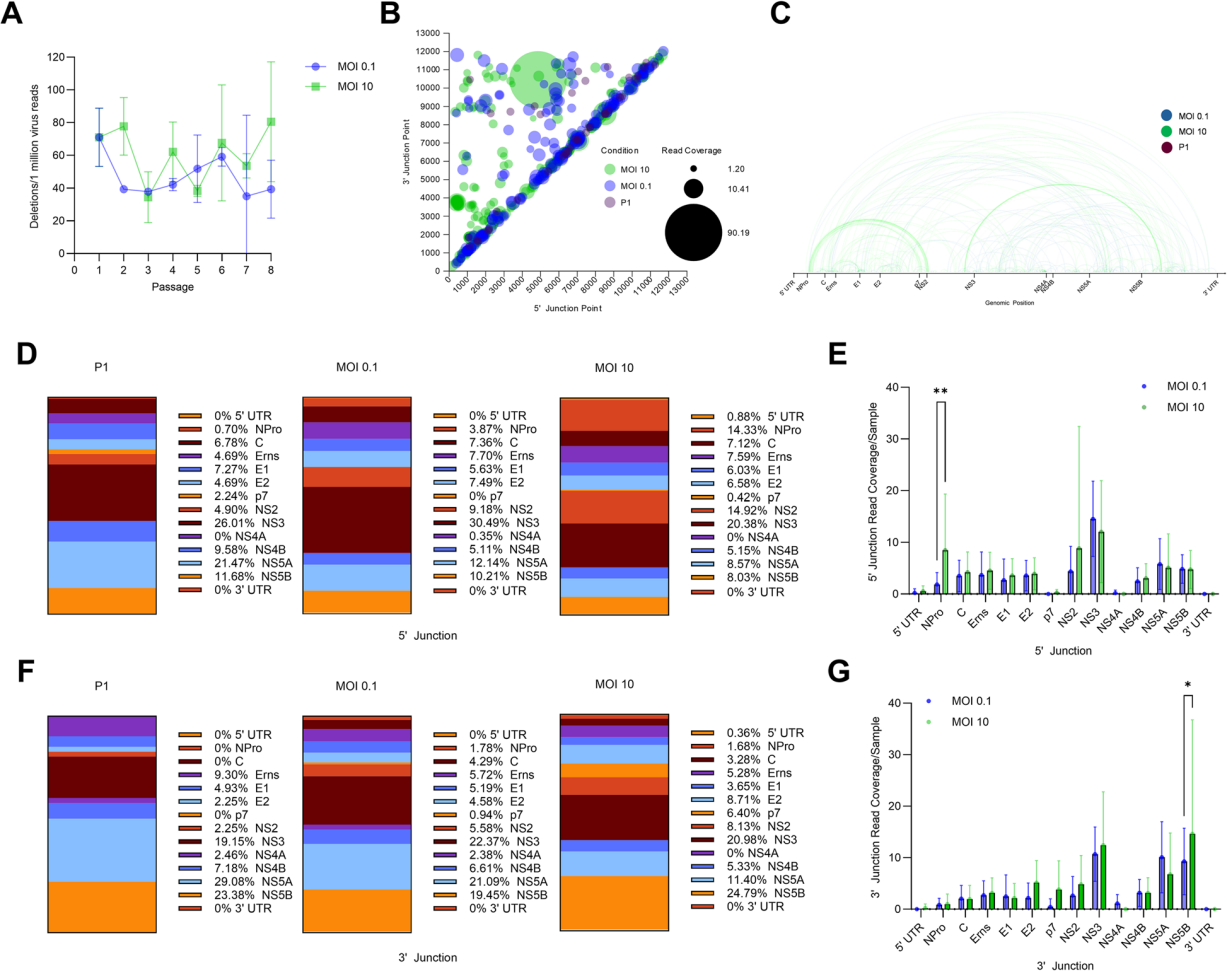
*In vitro* generation of DelVGs at different MOIs. BVDV1a cytopathic strain Singer plaques were purified and passaged at MOIs of 0.1 and 10 in BTu cells. RNA was extracted and sequenced via the Illumina MiSeq platform. NGS data were queried for NSVGs via the VODKA2 pipeline. **A** Total deletion reads (read coverage per 1 million standard viral reads) per passage at the two MOIs. **B** 5’ and 3’ junction point analysis for all BVDV1b strains. The area of each data point correlates to the coverage of DelVG reads per 1 million standard viral reads. **C** Representative depiction of the 5’ and 3’ junction points of individual DelVGs. Each arc represents an individual DelVG species. The width of each arc is proportional to read coverage (per 1 million standard viral reads) per DelVG species. Arcs are color coded: 0.1 MOI (blue), 10 MOI (green), Passage 1 (plum). Distribution of DelVG 5’ junction points (**D**) and 3’ junction points (**F**) per gene feature for passage 1, MOI 0.1 passages, and MOI 10 passages. Locations of the 5’ (**E**) and 3’ (**G**) junction points of all MOIs 0.1-passage and MOIs 10-passage Singer DelVG reads (read coverage per 1 million standard viral reads). Two-way ANOVA with Ŝídák multiple comparison test was performed for statistical analysis. Ns: p>0.05, *: P<0.05, **: p<0.005

We assessed whether the MOI affects the frequency and nature of DelVGs generated by BVDV by passing the BVDV1a Singer strain in BTu cells at two different MOIs (0.1 and 10) over 8 passages. Viral passage at both MOIs demonstrated a periodic increase and decrease in the generation of NSVGs over the course of the experiment, with the higher MOI condition having shorter intervals between high and low points (Fig. 5A). The periodic nature of NSVGs has been well characterized [57]. While both infection conditions generate short DelVGs throughout the entire length of the BVDV genome, infection with an MOI of 10 generated greater quantities of distinct DelVG species, specifically NPro-NS2, NPro-p7, and NS2-NS5B deletions. (Fig 5 B, C). In addition to more distinctive species, the 10 MOI infection condition generated more DelVG reads (Supplemental Fig. 2). These increases also translate to a larger set of common deletions observed across passages (Table 2). The deletion species generated by the 10 MOI condition presented different distributions in terms of the percentages of 5’ junction points and 3’ junction points within each gene feature (Fig 5D, F). A higher MOI increased the number of NPro and NS2 5’ junctions, whereas a lower MOI resulted in a greater percentage of NS3 and NS5 5’ junctions. The higher-MOI condition had a slightly greater percentage of p7 and NS5B 3’ junctions, whereas the lower-MOI condition had more 3’ junctions in the NS5 A region. Overall, the only significant difference between the low- and high-MOI infection conditions was an increase in NPro 5’ junction point reads and an increase in NS5B 3’ junction point reads (Fig 5E, G).

**Table 2:**
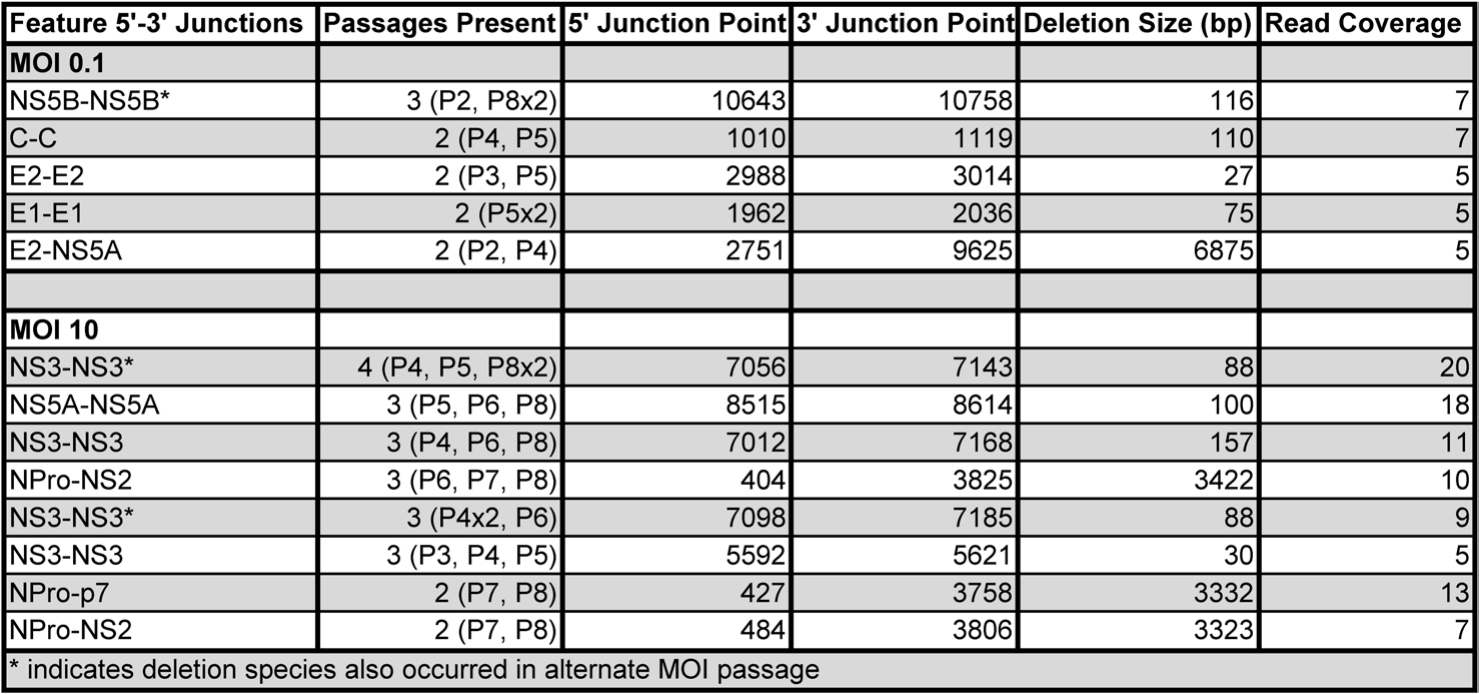
Most common Singer passage deletion species (n ≥2 samples)

Common DelVG species determined by presence in 2 or more passages of BVDV Singer at MOI 0.1 or MOI 10. From left to right: gene feature where 5' and 3' junctions occur; Number of passage samples DelVG species is present in (Which passage and number of replicates present in it); 5' junction point locus in genome; 3' junction point locus in genome; Size of DelVG in base pairs; Sum of read coverage across all passage samples, normalized per 1 million standard viral reads

## Discussion

This study reports the first characterization of NSVGs generated by BVDV or any *pestivirus* during viral replication. Synthetically engineered deletion mutants of BVDV have been generated and characterized previously [58, 59]. However, naturally occurring deletion mutations and their impact on the BVDV and *pestivirus* viral life cycles and evolution are not well understood. Therefore, this study increases the understanding of NSVG diversity and potential ramifications for BVDV. While negative-sense RNA viruses are known for generating both copyback CbVGs and deletion DelVGs, positive-sense RNA viruses, including *Flaviviridae*, primarily generate DelVG species and not CBVGs [18, 39–41]. The development and optimization of bioinformatic tools such as VODKA2, which can detect both CbVG and DelVG reads in NGS data, allows broad analysis of viral sequences [44]. While BVDV is highly diverse, with 2 genotypes, BVDV1 and BVDV2, which are currently divided into 21 subgenotypes (BVDV1a-u) and 4 subgenotypes (BVDV2a-d), we decided to focus this study on assessing the most prevalent historical and circulating subgenotypes BVDV1a and 1b [10].

In our assessment of more than 70 strains of BVDV1a and BVDV1b subgenotypes, like other positive-sense RNA viruses, DelVGs are generated during replication. Unsurprisingly, given the broad diversity of BVDV virus strains, a diverse set of DelVGs are generated in terms of the size of the deletions generated and where those deletions occur in the genome (Figs. 1, 2). Additionally, like other viruses, viral growth dynamics can impact DelVG generation in BVDV [20–22]. Using the BVDV1a Singer virus, growth conditions at two different MOIs can generate significantly different amounts of DelVGs and impact the DelVG species generated (Fig. 5). While certain gene features are hotspots for deletions, 5’ and 3’ junctions were found across the entire genome span in both BVDV1a and BVDV1b. Interestingly, striking differences emerged between the subgenotypes. While BVDV1a viruses generate a majority of DelVGs within the C region, only 8% of the 5’ junctions and 2% of the 3’ junctions of BVDV1b DelVGs occur within that region. A second major hotspot of BVDV1a deletions with 5’ junctions in the NS2 region and 3’ junctions in the NS5B region has also been reported (Fig. 1). Neither of these are major hotspots for BVDV1b deletions. For BVDV1b, the most prevalent hotspot for DelVGs is the 5’ junction in NS4B and the 3’ junction in NS5 A (Fig 2), accounting for a third of the BVDV1b DelVG reads. While there are distinct differences between subgenotypes, within each group, there was much less of a phylogenetic link between the deletion species and closely related BVDV1 strains. While there are a few small clusters of strains with increased DelVG reads and reads per DelVG species, for BVDV1a (Fig. 1), overall, there are few DelVG species with size or quantity differences within each subgenotype. Surprisingly, this trend also translates to common DelVG species. Only 4 of the 15 (27%) DelVG species present in at least 3 viral strains demonstrated any kind of phylogenetic clustering. The most common DelVG species were broadly distributed along the subgenotypic phylogenetic trees (Fig 3). These results suggest an evolutionary divergence of DelVGs between BVDV1a and BVDV1b, whereas within each subgenotype, there is a weaker correlation with genetic differences.

In the broad side-by-side comparison of subgenotypes, BVDV1a generated significantly more deletions than did BVDV1b, with this increase being directly related to significantly more DelVGs with deletions in the C region (Fig. 4). The BVDV C protein is a small, highly basic, disordered structural protein with minimal secondary structure. While its role in viral assembly is poorly understood, like other viral capsids, it has RNA-binding properties [60]. Other flavivirus C proteins have been studied more thoroughly and have been found to play numerous roles, including interactions that impact host RNA splicing and transcription, sequestration of short interfering (si)-RNAs, and being crucial for efficiently packaging viruses during assembly [61–63]. Deletions in the C region of flaviviruses have been linked to viral attenuation and are an active area of vaccine development [64–66]. In these studies, various deletion sizes and loci in the C region can lead to varying degrees of attenuation. Notably, the capsid can withstand many of these deletions and still package recoverable viruses [65]. In the case of the BVDV1a strains queried, over half of the DelVG reads had 5’ and/or 3’ junctions in C. Whether the C region from any of these DelVG genomes is functional for viral packaging or attenuated in any way is unknown. Notably, while more than half of the DelVG reads for BVDV1a viruses have deletions within the C region, those for BVDV1b are less than 10%. Over the last 35 years, the prevalent subtype of circulating BVDV1 has shifted from BVDV1a to BVDV1b, with 51% of BVDV1 being 1a in 1988, to accounting for only 18% of BVDV1 viruses as of 2008 [67, 68]. Further study is needed to understand the impact and dynamics of these DelVGs, but the generation of these C deletions may contribute to the reduction in circulating BVDV1a and increase in circulating BVDV1b viruses.

NSVGs play critical roles during the life cycle of RNA viruses. NSVGs have been characterized for both positive- and negative-sense viruses [18]. While not previously reported to be strong inducers of innate immunity, such as CbVGs, DelVGs have been associated with the establishment of viral persistence [18, 24, 25]. BVDV persistently infects animals and remains a primary concern of the livestock industry, causing a significant economic impact [4–7]. Differences in DelVG profiles may contribute to the establishment of viral persistence during transplacental infection and the natural selection of fitter viral variants. While persistence is attributed to the NCP biotype of BVDV, it was surprising that there were no significant differences between the 5 pairs of NCP and CP viruses queried in terms of the number of DelVGs or species breakdown (Fig. 4) [69, 70]. A broader query of NCP and CP viruses from this virus set was not conducted because the data were imbalanced for biotype and not representative. Future studies will expand upon these biotype differences. In addition to being a complex part of viral replication and host‒virus interactions, NSVGs are an active area of vaccine and antiviral development [31–34].

## Conclusions

In this study, we reported the first characterization of deletion viral genomes generated during the replication of a pestivirus. Here, we describe a diverse set of DelVGs generated by BVDV1a and BVDV1b and detail the differences between the two subgenotypes. The role of DelVGs in virus‒host interactions, viral evolution, and the establishment of the persistence of BVDV is unknown but may provide key insights into better understanding the virus, elucidating new antiviral targets, and generating better vaccines.

## Supplementary Information

Supplementary Material 1.

## Data Availability

Genome sequences for all strains were deposited to the GenBank database. Accession numbers are available in supplemental table 1.

## Abbreviations

BRDC: Bovine respiratory disease complex,
BVDV: Bovine viral diarrhea virus
BTu: Bovine turbinate cells
C: Capsid/core
CbVG: Copyback viral genome
CP: Cytopathic
CPE: Cytopathic effect
DIP: Defective interfering particle
DVG: Defective viral genome
DelVG: Deletion viral genome
MOI: Multiplicity of infection
MDBK: Madin-Darby bovine kidney cells
NCP: Noncytopathic
NGS: Next-generation sequencing
NSVG: Nonstandard viral genome
ORF: Open reading frame
PCR: Polymerase chain reaction
RdRp: RNA-dependent RNA polymerase
UTR: Untranslated region
VODKA2: Viral opensource DVG Key Algorithm 2
