# Supplementary material for: Deletion viral genome diversity among bovine viral diarrhea virus (BVDV) 1a and 1b strains
Source: Virol J. 2025 Jul 14;22:237. doi: 10.1186/s12985-025-02773-z (PMC12257715; doi:10.1186/s12985-025-02773-z)

**Supplemental Materials**

**Supplemental Figure 1.** Phylogenetic dendrograms of the BVDV1a (**A**) and BVDV1b (**B**) strains generated via Clustal W alignment and the UPGMA method via MEGA7 software. Branch support was estimated via 1000 bootstrap replicates and the Poisson correction method to calculate the evolutionary distances. Verified comparative sequences from NCBI GenBank; BVDV1a (green), BVDV1b (orange), BVDV2a (blue).


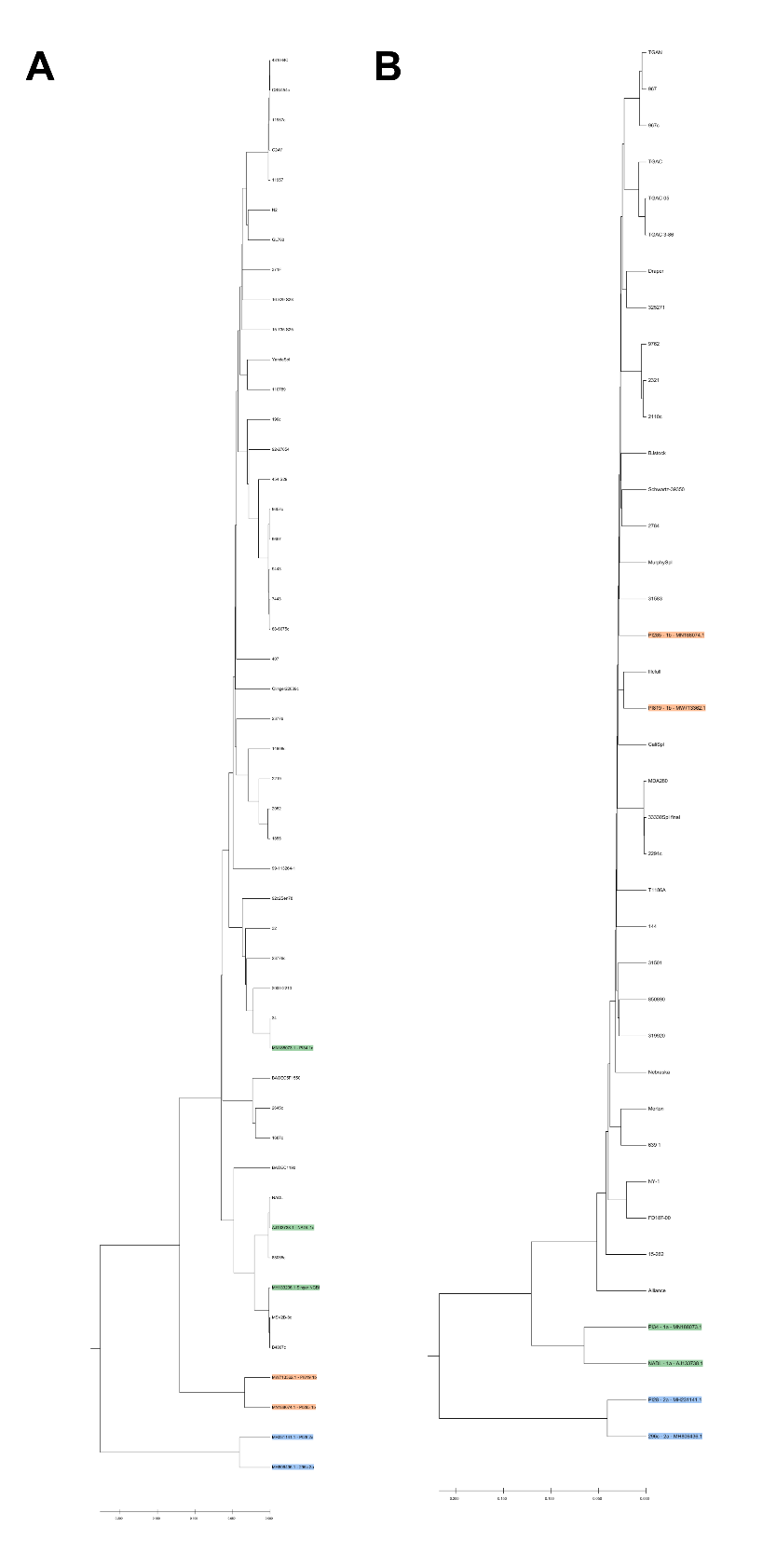


**Supplemental Figure 2.** Total deletion reads (read coverage per 1 million standard viral reads) among BVDV1a Singer passaged at MOIs of 0.1 (blue) and 10 (green). A paired (passage number) t test was performed for statistical analysis. *: p<0.05.

**
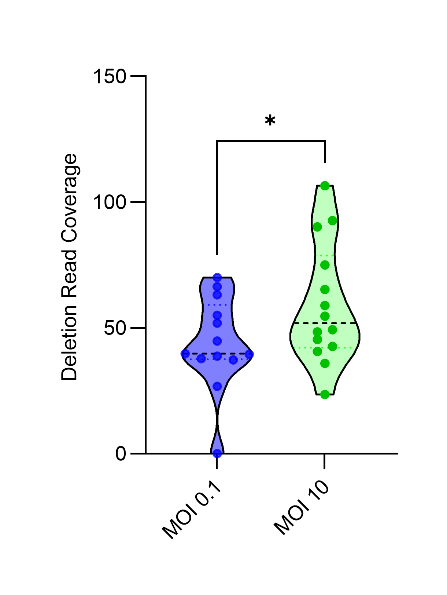
**

**Supplemental Table 1.**


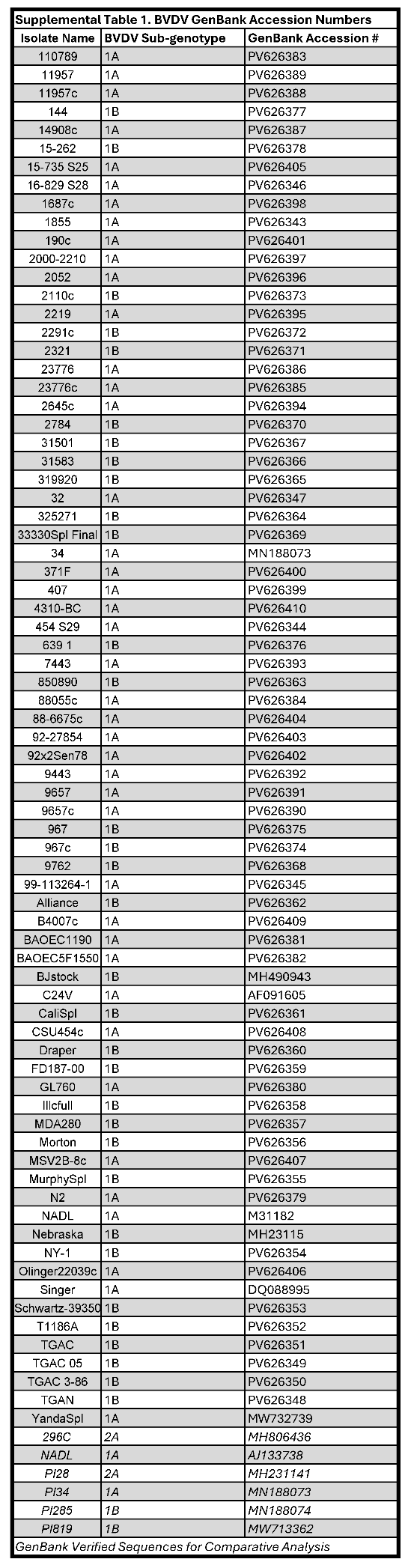

Supplement: Supplementary file 1 — Supplementary Material 1. [file 12985_2025_2773_MOESM1_ESM.docx]
